# Peripapillary and macular microvasculature features of non-arteritic anterior ischemic optic neuropathy

**DOI:** 10.3389/fmed.2022.1033838

**Published:** 2023-01-12

**Authors:** Sangeethabalasri Pugazhendhi, Miaomiao Yu, Gabriella Zhou, Yuxuan Chen, Ruikang Wang, Yaping Joyce Liao

**Affiliations:** ^1^Department of Ophthalmology, School of Medicine, Stanford University, Stanford, CA, United States; ^2^Department of Bioengineering, University of Washington, Seattle, WA, United States; ^3^Department of Ophthalmology, University of Washington, Seattle, WA, United States; ^4^Department of Neurology, School of Medicine, Stanford University, Stanford, CA, United States

**Keywords:** non-arteritic anterior ischemic optic neuropathy (NAION), optical coherence tomography (OCT), optical coherence tomography angiography (OCTA), superficial capillary plexus (SCP), retinal nerve fiber layer (RNFL), ganglion cell complex (GCC)

## Abstract

**Purpose:**

The hallmark of non-arteritic anterior ischemic optic neuropathy (NAION) is vascular compromise to the anterior optic nerve and thinning of the retinal nerve fiber layer (RNFL) and secondary degeneration of the retinal ganglion cell body or thinning of the ganglion cell complex (GCC). This study investigates optical coherence tomography (OCT) and OCT Angiography (OCTA) changes in chronic NAION and identifies imaging biomarkers that best predict disease.

**Methods:**

We performed a retrospective case-control study of 24 chronic NAION eyes (18 patients) and 70 control eyes (45 patients) to compare both whole-eye and regional OCT, OCTA, static perimetry measurements. OCT measurements were quantified automatically using commercial software, and OCTA was analyzed using custom MATLAB script with large vessel removal to measure 154 total parameters per eye.

**Results:**

We confirmed that static perimetry mean deviation (MD) was significantly worse in chronic NAION (–13.53 ± 2.36) than control (–0.47 ± 0.72; *P* < 0.001) eyes, and NAION eyes had 31 μm thinner RNFL (control: 95.9 ± 25.8 μm; NAION: 64.5 ± 18.0, *P* < 0.001), and 21.8 μm thinner GCC compared with controls (control: 81.5 ± 4.4 μm; NAION: 59.7 ± 10.5, *P* < 0.001). Spearman correlation analysis of OCTA parameters reveal that vessel area density (VAD) and flux are highly correlated with visual field MD and OCT measurements. Hierarchical clustering two distinct groups (NAION and control), where standardized measurements of NAION eyes were generally lower than controls. Two-way mixed ANOVAs showed significant interaction between patient status (control and chronic NAION) and structure (optic disk and macula) for annulus VAD and flux values and mean RNFL and GCC thickness. *Post-hoc* tests showed this effect stems from lower peripapillary values in NAION compared to controls. Separate logistic regression models with LASSO regularization identified VAD and flux are one of the best OCTA parameters for predicting NAION.

**Conclusion:**

Ischemic insult to the optic disk is more severe likely from primary degeneration of the affected peripapillary region while macula is affected by secondary retrograde degeneration and loss of retinal ganglion cells. In addition to OCT measurements, peripapillary and macular vascular parameters such as VAD and flux are good predictors of optic nerve and retinal changes in NAION.

## Introduction

Non-arteritic anterior ischemic optic neuropathy (NAION) is a common cause of vision loss that typically occurs in patients older than 50 years of age (1–3). NAION occurs due to ischemia of the anterior part of the optic nerve, which is supplied by the posterior ciliary artery (4). Although the pathophysiology of NAION is multifactorial and unclear, several risk factors have been identified including systemic hypertension, diabetes mellitus, obstructive sleep apnea (OSA) and other optic disk abnormalities (5–7).

Non-arteritic anterior ischemic optic neuropathy is clinically characterized by acute onset of painless loss of vision with a relative afferent pupillary defect with generalized or sectoral optic disk edema, which may or may not be accompanied by peripapillary hemorrhages in fundoscopic examination (8, 9). The optic disk edema typically disappears within a few weeks and is usually followed by optic disk pallor in the chronic stage of NAION. Chronic NAION is typically characterized by resolved optic disk edema with an improvement in visual acuity, but persistence of visual field loss and optic disk pallor due to ischemic damage to the retinal ganglion cells and ganglion cell loss (10–12). Recent advances in ophthalmic imaging technology have contributed significantly in elucidating the pathophysiology of NAION and improving its diagnosis and management.

Optical coherence tomography angiography (OCTA) is a promising new non-invasive ophthalmic imaging technology for visualizing and quantifying both peripapillary and parafoveal microvasculature (13–15). OCTA imaging can visualize retinal vessels with the detection of motion contrast from the blood flow through the Superficial Capillary Plexus (SCP), which is present between the Internal Limiting Membrane (ILM) and Inner Plexiform Layer (IPL). Visual field loss in NAION is thought to arise from decreased perfusion to the anterior optic nerve and surrounding structures, leading to a reduction in the vascular density of the SCP in the peripapillary region and axonal degeneration in the acute stage of NAION (2, 14, 16–20). Over time, NAION results in secondary loss of SCP density in both the peripapillary and parafoveal region (21–25). In this study, we analyze the vascular, structural, and functional parameters using OCTA, OCT, and paired static perimetry measurements of the peripapillary and macular region in chronic NAION patients and age-matched controls to determine which parameters are most correlated with vision loss. By identifying these key correlations and biomarkers, non-invasive imaging like OCTA can be used to determine changes in the peripapillary and parafoveal microvasculature that occurs due to primary and secondary ischemic insults of NAION, and to predict the risk of vision loss in NAION.

## Materials and methods

### Participants and clinical evaluation

We performed a retrospective cross-sectional study of patients with chronic NAION and controls who were evaluated at Byers Eye Institute at Stanford University Medical Center between July 2020 and May 2021. This study was approved by the Institutional Review Board by Stanford University and adhered to the Declaration of Helsinki and the Health Insurance Portability and Accountability Act.

#### Inclusion/exclusion

**FIGURE 1:**
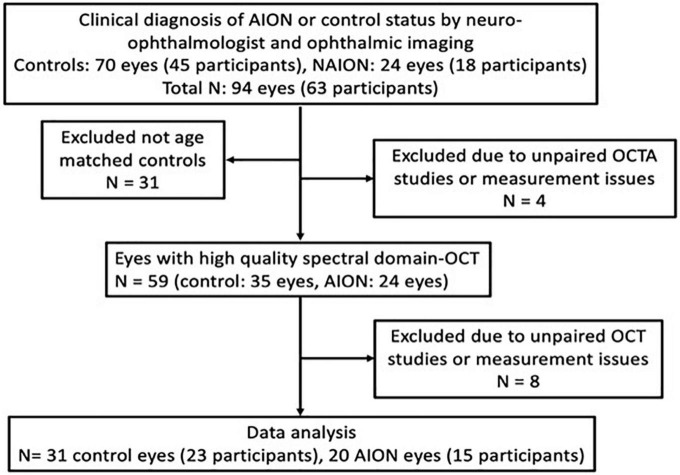
Patient selection flow chart for chronic non-arteritic anterior ischemic optic neuropathy (NAION) (≥6 months from onset) and healthy controls.

Clinical diagnosis of NAION with confirmation in the acute setting by neuro-ophthalmologist or fundus imaging. In total, we enrolled 63 participants (94 eyes) retrospectively with a clinical diagnosis of NAION (24 eyes) or control status (70 eyes) by neuro-ophthalmologist and ophthalmic imaging (Figure 1). Control eyes (*n* = 31) that were not age-matched were excluded. These patients were then narrowed to include only those who had high-quality swept-source OCTA (PLEX^®^ Elite 9,000; Carl Zeiss Meditec, Inc., Dublin, CA, United States) and spectral-domain OCT (Carl Zeiss Meditec Inc., Jena, Germany). We excluded all patients in acute stage of disease, patients with optic neuropathy other than chronic stage of NAION and those with ophthalmic, or systemic diseases that may affect the OCT and OCTA measurements.

In the NAION group, four eyes were excluded because of lack of paired high quality peripapillary and macular OCT or OCTA images, and in the control group, eight eyes were excluded due to lack of supplementary OCT measurements or lack of paired peripapillary and macular images. In total, we included 15 patients (20 eyes) with chronic NAION and 23 controls (31 eyes). All NAION subjects had comprehensive neuro-ophthalmic examination by one investigator (YJL) and confirmation by static perimetry, color and autofluorescence fundoscopic photography and OCT imaging. All subjects also had comprehensive ophthalmic measurements including best corrected visual acuity (BCVA) using the Snellen chart to calculate the logarithm of reciprocal decimal visual acuity (logMAR), refraction, intraocular pressure measurement and ophthalmoscopy. Chronic NAION patients were characterized by patients with the last episode more than 6 months prior to OCTA image acquisition. In order to have controls with similar demographics as the NAION patients, we included controls of age ≥45 years. The eligibility criteria for control subjects include having BCVA of equal or better than logMAR VA 0.2 and normal optic nerves per fundus examination, no subjective visual field loss and normal retinal nerve fiber layer (RNFL) and ganglion cell complex (GCC) OCT measurements. Control eyes that did not meet the eligibility criteria were excluded from the study, which resulted in the use of only one in many control patients with no ocular or systemic diseases. Four eyes were contralateral control eyes from patients with unilateral NAION. In addition, eight eyes were contralateral control eyes in patients with unilateral unrelated pathology.

### Static perimetry measurements and analysis

Automated static perimetry was performed using Humphrey Field Analyzer (Carl Zeiss Meditech, Inc., Dublin, CA, United States) using the standard 24-2 Swedish Interactive Thresholding Algorithm fast strategy. The mean deviation (MD) was automatically calculated by the Humphrey Field Analyzer. We excluded unreliable visual field tests, which were defined as fixation loss >20% or false-positive or false-negative error rates >20%.

To perform our Spearman correlation analysis, the topographical visual field MD data was translated to match the topographic location of the OCTA regions. For example, supero-temporal region of visual field with a defect would be translated to infero-nasal region to match the presentation in the OCTA images. This OCT-centric data of visual field MD and OCTA correlation provides more clarity for data presentation and interpretation of results.

Sectorial division of the peripapillary region was performed using modified Garway-Heath map regionalization. According to the Garway-Heath map, the sectors are named according to their location on the optic nerve, and not according to their location in the visual field to allow for detailed comparison of structural and functional measurements (26). The temporal disk sector corresponds to the nasal sector of the visual field, and vice versa for nasal disk sector. The supero-nasal disk sector corresponds to a peripheral, arcuate shaped sector in the infero-temporal and infero-nasal visual field, and vice versa for infero-nasal disk sector. The supero-temporal disk sector corresponds to a paracentral, arcuate shaped sector in the infero-temporal and infero-nasal visual field, and vice versa for infero-temporal disk sector.

### OCT and OCTA acquisition and analysis

Spectral-domain OCT images were acquired using Cirrus HD-OCT (Carl Zeiss Meditec Inc., Jena, Germany) using a light source of 840 nm wavelength. The instrument has maximum A-scan speed of 68,000 scans/s with optical axial resolution of 5 μm and scanning depth of 2 mm. We performed the Optic Disk Cube 200 × 200 acquiring 200 horizontal scan lines each composed of 200 A-scans and Macular Cube 512 × 128 scans acquiring 128 horizontal scan lines each composed of 512 A-scans. The thickness of the RNFL was automatically measured in a circle with 3.46 mm diameter centered on the optic disk. The thickness of the macular GCC was measured in an elliptical annulus with a vertical inner and outer radius of 0.5 and 2.0 mm, and horizontal inner and outer radius of 0.6 and 2.4 mm, respectively, around the fovea which consists of the combined thickness of the ganglion cell layer and inner plexiform layers.

Swept-source OCTA images were acquired using Plex ELITE 9000 (Carl Zeiss Meditec, Inc., Dublin, CA, United States) using a light source between 1,040 and 1,070 nm. The instrument has a maximum scan speed of 200,000 A-scans/s with an optical axial resolution of 1.95 μm and scanning dept of 3.0 mm. The 6 × 6 mm^2^ square angiography scans of the optic disk and the macula were obtained using the FastTrac eye tracking system. Automatic segmentation of the raw en face OCTA data was performed using in-unit optic microangiography (OMAG). Only images with signal strength index >7 were used for analysis. We used a customized quantification software (coded with MATLAB R2020b; MathWorks, Natick, MA, United States) to quantify OCTA images based on modification of previous algorithm, which measures six vascular parameters of the peripapillary and parafoveal region including flux, vessel area density (VAD), vessel skeleton density (VSD), vessel perimeter index (VPI), vessel complexity index (VCI), and vessel diameter index (VDI) (27). In brief, the validated algorithm processes the original OCTA image into a binary vessel map using a combined three-way method involving global thresholding, Hessian filter and adaptive threshold. In addition, vessel skeleton map was created by linearizing every vessel, regardless of its size or diameter, into single-pixel width to obtain vessel length information. Vessel perimeter map was created by mapping the edges of the vessels to obtain information about vessel perimeter. All vascular quantitative parameters were calculated based on the above mentioned three vessel maps (27). Furthermore, we removed the large vessels from the images and analyzed an annulus with an inner diameter of 0.5 mm and outer diameter of 2.4 mm around the disk and macula.

**FIGURE 2:**
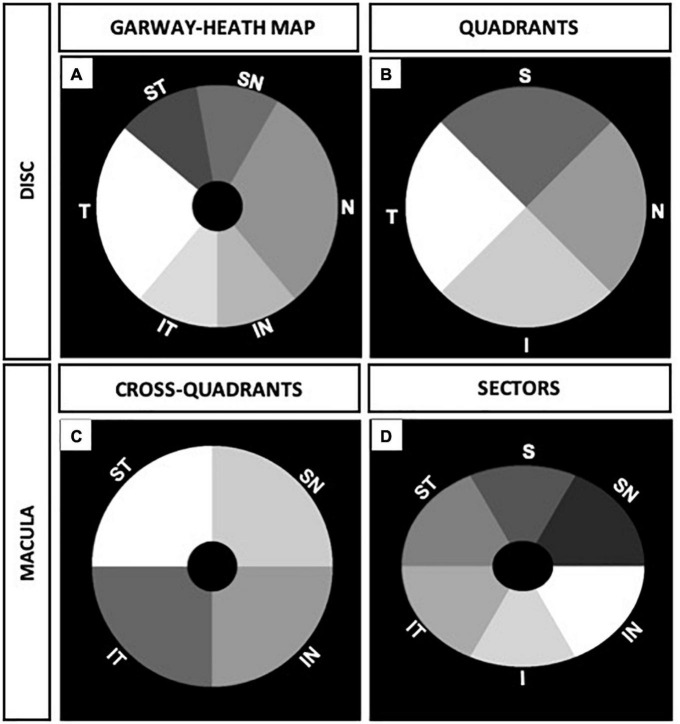
Peripapillary and macular annulus masks of right eye. **(A)** Six sections of optic disk corresponding to modified visual field Garway-Heath map. **(B)** Four optic disk quadrants corresponding to retinal nerve fiber layer (RNFL) quadrants from optical coherence tomography (OCT) measurements. **(C)** Four cross-quadrants of macula corresponding with Visual Field quadrants. **(D)** Six macular sectors in ellipsoid annulus corresponding to ganglion cell complex (GCC) sectors from OCT measurements.

For the optic disk, the OCTA parameters in sections according to modified Garway-Heath map were measured using the quantification software as shown in the mask in Figure 2A for correlation with visual field MD. The peripapillary OCTA parameters of four quadrants resembling the RNFL quadrants from OCT imaging were measured as shown in Figure 2B for correlation with RNFL thickness measurements on OCT.

For the macula, OCTA parameters were divided into four cross quadrants (supero-nasal, supero-temporal, infero-nasal, and infero-temporal cross-quadrants) and analyzed as shown in Figure 2C for functional correlation with the visual field quadrants MD. In addition, we analyzed six sectors of the macula (resembling GCC sectors from OCT imaging) using an elliptical annulus with a horizontal inner and outer diameter of 0.6 and 2.4 mm, and vertical inner and outer diameter of 0.5 and 2.0 mm, respectively. The parafoveal OCTA parameters in sectors were measured as shown in the mask in Figure 2D for structural correlation with OCT GCC measurements.

### Statistical analysis

The data were analyzed using Python programming language (Python Software Foundation, version 3.9.6)^1^ and R (R Foundation for Statistical Computing, Vienna, Austria). Parameters were quantified using mean and standard deviation (SD) or median and 95% confidence interval. Age and static perimetry MD of control and patient groups were analyzed using Student’s *t*-test and Mann-Whitney U tests; gender was analyzed using chi-square test. OCTA vessel parameter measurements were correlated with static perimetry MD and OCT measurements using Spearman correlation tests. Correlation coefficients between 0.7–1, 0.3–0.7, and 0–0.3 are rated as high, moderate, and low, respectively.

## Results

### Peripapillary and macular microvascular dropout corresponds with thinning on OCT and reduce static perimetry in chronic NAION

To identify novel vascular biomarkers of chronic NAION, we conducted a cross-sectional study of visual function and structure using 20 eyes of 15 chronic NAION patients (33% bilateral, 67% unilateral; age 62.7 ± 2.1 years) and 31 eyes from 23 controls (age 59.2 ± 1.7 years). Only two control eyes are contralateral unaffected eyes of NAION patients. In the NAION group, four eyes were excluded because there were no paired high quality peripapillary and macular images. In the control group, eight eyes were excluded due to lack of supplementary OCT measurements or lack of paired peripapillary and macular images (see section “Materials and methods”). As expected, visual function and optic nerve structure were worse in NAION eyes compared with control eyes (Table 1). NAION eyes had worse logMAR (control: 0.0; NAION: 0.1, *P* < 0.126) and significantly worse static perimetry mean deviation than control eyes (control: –0.5 ± 0.7; NAION: –13.5 ± 2.4, *P* < 0.001) (Table 1). Structural measurement using sd-OCT revealed the NAION eyes had 31 μm thinner RNFL (control: 95.9 ± 25.8 μm; NAION: 64.5 ± 18.0, *P* < 0.001) and 21.8 μm thinner GCC compared with controls (control: 81.5 ± 4.4 μm; NAION: 59.7 ± 10.5, *P* < 0.001) (Table 1).

**TABLE 1:** Study population demographics of control and patient (eyes) with chronic non-arteritic anterior ischemic optic neuropathy (NAION).

	Control	NAION	
Summaries	31 eyes (23 subjects)	20 eyes (15 subjects)	–
Gender	Male: *n* = 14 (45.2%) Female: *n* = 17 (54.8%)	Male: *n* = 13 (65.0%) Female: *n* = 7 (35.0%)	X^2^ (1) = 1.92, *p* = 0.166
Age (years)	59.2 ± 1.7 years	62.7 ± 2.1 years	Welch t (39.89) = 1.98, ***p* = 0.054**
Eyes	OD: *n* = 18 (58.1%) OS: *n* = 13 (41.9%)	OD: *n* = 8 (40.0%) OS: *n* = 12 (60.0%)	X^2^ (1) = 1.59, *p* = 0.208
Static perimetry (MD)	-0.5 ± 0.7	-13.5 ± 2.4	Mann-Whitney U = 7.00, ***p* < 0.001**
LogMAR	0.0, IQR = 0.1	0.10, IQR = 0.2	Mann-Whitney U = 243.00, *p* = 0.126
RNFL (μm)	95.9 ± 25.8	64.5 ± 18.0	Welch t (226.08) = -10.52, *p* < 0.001
GCC (μm)	81.3 ± 4.9	59.7 ± 10.5	Welch t (175.18) = -22.02, *p* < 0.001

Bold values represent the significance.

**FIGURE 3:**
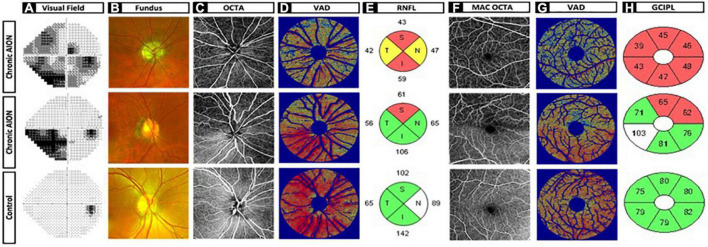
Chronic non-arteritic anterior ischemic optic neuropathy (NAION) optic disk and macula images and heatmaps versus control. **(A)** Humphrey visual field. **(B)** Fundus imaging. **(C)** OCTA optic disk 6 × 6 original image. **(D)** Vessel area density heatmap of optic disk. **(E)** Retinal nerve fiber layer quadrants. **(F)** OCTA macula 6x6 original image. **(G)** Vessel area density heatmap of macula. **(H)** Ganglion cell complex inner plexiform layer sectors. Row 1: Patient 1 is a representative 63-year-old woman with generalized visual field loss mean deviation (MD –14.94 dB), generalized thinning of retinal nerve fiber layer (RNFL), and ganglion cell complex (GCC) (48 and 45 mm, respectively), and diffuse vascular dropout in the peripapillary and macular SCP. Row 2: Patient 2 is a 69-year-old woman with inferior altitudinal moderate visual field loss (MD –10.90 dB), superior thinning of RNFL and GCC (72 and 76 mm, respectively), and superior drop out of peripapillary (Figure 2B, middle row) and macular SCP vasculature. Row 3: Patient 3 is a 58-year-old woman as the representative control patient with no visual field loss (MD 0.08 dB), no thinning of RNFL and GCC (100 and 79 mm, respectively), and dense peripapillary and macular microvasculature with no vessel dropout.

We used ss-OCT to image peripapillary and macular superficial capillary plexus (SCP), which reflect the most important vascular changes in optic neuropathies such as NAION, because this is the current best technology due to higher resolution, better en face images, reduced eye movement artifact, and faster image acquisition (28–30). Comparison of 6 × 6 mm SCP in NAION and controls revealed different patterns of vascular dropout that correspond to RNFL and GCC thinning on sd-OCT and visual field defect on static perimetry, as shown in Figure 3. Patient 1 is a representative 63 years-old woman with generalized visual field loss (MD –14.94 dB), generalized thinning of RNFL and GCC (48 and 45 μm, respectively), and diffuse vascular dropout in the peripapillary and macular SCP. Patient 2 is a 69 years-old woman with inferior altitudinal moderate visual field loss (MD –10.90 dB), superior thinning of RNFL and GCC (72 and 76 μm, respectively), and superior drop out of peripapillary (Figure 2B, middle row) and macular SCP vasculature (Figure 3).

Using custom MATLab algorithm, we analyzed peripapillary and macular SCP OCTA in all NAION and control eyes using the OMAG algorithm and calculated six microvascular parameters: VAD, VSD, VCI, VPI, VDI, and flux (27). To standardize the region of interest (ROI) around the peripapillary area, we used an annulus around the center of the optic disk (outer diameter 2.4 mm, inner diameter 0.5 mm). For topographical quadrant analysis, we used a circle of radius 2.4 mm at the center of the optic disk. Annulus was not used in the quadrant analysis of the optic disk in order to simulate what is done for the RNFL analysis (Figures 2B, 3E). To standardize the ROI in the macula, we used an annulus circle (outer diameter 2.4 mm, inner diameter 0.5 mm) centered at the foveal avascular zone (FAZ). An elliptical annulus centered at the FAZ (vertical inner and outer diameter of 0.5 and 2.0 mm, and horizontal inner and outer radius of 0.6 and 2.4 mm) was used for sectoral analysis to simulate what is done for the GCC analysis in sd-OCT. To remove large vessel contribution, which is particularly critical in the peripapillary region, we first thresholded the 6 × 6 mm ss-OCTA images and removed large vessels, before calculating all OCTA measurements.

Comparing to control eyes, we found that every ss-OCTA parameter was generally lower in chronic NAION (Tables 2, 3). Peripapillary VAD, the most commonly calculated OCTA value in commercial instruments (27, 31), was significantly decreased in NAION by 15% when compared to the mean peripapillary annulus VAD value of controls (Tables 2, 3). Mean annulus peripapillary flux, which is the number of blood cells passing through the retinal vessel cross-sectional area per unit time, was decreased by 29% in NAION compared to that of controls. The remaining four peripapillary mean annulus vascular parameters were also significantly decreased in NAION eyes compared to controls but to a lesser degree than flux: VSD and VCI by 9%, VPI by 10%, and VDI by 5% (Table 2). In our study, macular mean annulus VAD and flux were significantly decreased in NAION by 10 and 21%, respectively, when compared to the mean macular annulus VAD and flux value of controls (Table 3). Resembling the pattern of peripapillary values, the remaining four macular annulus parameters were also significantly decreased in NAION eyes when compared to mean annulus values of control but to a lesser degree than VAD and flux: VSD by 5%, VCI by 6%, VPI by 7%, and VDI by 3% (Table 3).

**TABLE 2:** Differences in peripapillary annulus and regional optical coherence tomography angiography (OCTA) parameters between controls and eyes with chronic non-arteritic anterior ischemic optic neuropathy (NAION).

	Control	NAION	*P*-value
**VAD**			
Annulus	0.60 ± 0.02	0.51 ± 0.03	**9.31E-10**
Supero-temporal	0.66 ± 0.02	0.57 ± 0.04	**6.29E-06**
Supero-nasal	0.64 ± 0.02	0.55 ± 0.06	**5.24E-05**
Infero-temporal	0.67 ± 0.02	0.6 ± 0.06	**9.47E-05**
Infero-nasal	0.64 ± 0.03	0.57 ± 0.06	**4.20E-04**
Temporal	0.63 ± 0.02	0.58 ± 0.03	**1.70E-04**
Nasal	0.61 ± 0.02	0.54 ± 0.06	**9.70E-04**
**Flux**			
Annulus	0.62 ± 0.05	0.44 ± 0.07	**2.28E-09**
Supero-temporal	0.70 ± 0.06	0.43 ± 0.09	**6.29E-06**
Supero-nasal	0.64 ± 0.07	0.41 ± 0.10	**2.84E-05**
Infero-temporal	0.71 ± 0.07	0.52 ± 0.14	**7.79E-05**
Infero-nasal	0.63 ± 0.08	0.44 ± 0.11	**4.20E-04**
Temporal	0.66 ± 0.06	0.52 ± 0.09	**2.00E-04**
Nasal	0.56 ± 0.05	0.40 ± 0.10	**9.47E-05**
**VSD**			
Annulus	0.23 ± 0.01	0.21 ± 0.01	**8.08E-08**
Supero-temporal	0.23 ± 0.01	0.21 ± 0.02	2.50E-03
Supero-nasal	0.23 ± 0.01	0.21 ± 0.02	3.40E-03
Infero-temporal	0.24 ± 0.01	0.21 ± 0.02	3.90E-03
Infero-nasal	0.23 ± 0.01	0.21 ± 0.02	**8.20E-04**
Temporal	0.23 ± 0.01	0.23 ± 0.01	2.50E-03
Nasal	0.22 ± 0.01	0.21 ± 0.01	**6.40E-05**
**VCI**			
Annulus	9934.35 ± 414.13	9033.82 ± 435.85	**5.42E-08**
Supero-temporal	1070.86 ± 70.25	925.55 ± 82.17	**1.60E-04**
Supero-nasal	1064.40 ± 66.69	1000.46 ± 78.78	0.01
Infero-temporal	1072.07 ± 64.20	983.27 ± 82.39	4.50E-03
Infero-nasal	1019.42 ± 62.48	987.25 ± 128.22	0.26
Temporal	2652.46 ± 120.06	2468.29 ± 145.82	1.60E-03
Nasal	2948.82 ± 209.54	2717.65 ± 198.15	4.50E-03
**VPI**			
Annulus	0.50 ± 0.01	0.45 ± 0.02	**7.53E-09**
Supero-temporal	0.45 ± 0.03	0.40 ± 0.04	2.50E-03
Supero-nasal	0.44 ± 0.02	0.41 ± 0.05	7.00E-02
Infero-temporal	0.45 ± 0.02	0.39 ± 0.05	**2.00E-04**
Infero-nasal	0.45 ± 0.02	0.40 ± 0.02	**5.24E-05**
Temporal	0.47 ± 0.02	0.45 ± 0.03	2.00E-02
Nasal	0.45 ± -0.02	0.41 ± 0.01	**3.49E-05**
**VDI**			
Annulus	18.01 ± 0.53	17.08 ± 0.44	**1.20E-07**
Supero-temporal	19.62 ± 1.06	19.47 ± 0.77	0.39
Supero-nasal	19.32 ± 0.99	18.46 ± 0.67	0.03
Infero-temporal	19.41 ± 0.77	19.39 ± 0.65	0.49
Infero-nasal	19.92 ± 0.98	18.98 ± 0.96	0.01
Temporal	18.54 ± 0.74	18.15 ± 0.54	0.05
Nasal	19.14 ± 0.95	18.66 ± 0.82	0.08

Bold values denote statistical significance at the *p* < 0.05 level.

**TABLE 3:** Differences in macular annulus and cross-quadrant optical coherence tomography angiography (OCTA) parameters between controls and eyes with chronic non-arteritic anterior ischemic optic neuropathy (NAION).

	Control	NAION	*P*-value
**VAD**			
Annulus	0.52 ± 0.01	0.47 ± 0.03	**8.50E-09**
Supero-temporal	0.56 ± 0.01	0.53 ± 0.03	**6.80E-04**
Supero-nasal	0.58 ± 0.01	0.55 ± 0.03	0.01
Infero-temporal	0.56 ± 0.01	0.52 ± 0.02	**4.53E-05**
Infero-nasal	0.58 ± 0.02	0.54 ± 0.03	**6.80E-04**
**Flux**			
Annulus	0.48 ± 0.04	0.38 ± 0.05	**3.59E-08**
Supero-temporal	0.46 ± 0.03	0.38 ± 0.05	**2.50E-04**
Supero-nasal	0.51 ± 0.05	0.42 ± 0.06	**4.60E-04**
Infero-temporal	0.44 ± 0.05	0.35 ± 0.03	**1.40E-04**
Infero-nasal	0.50 ± 0.05	0.41 ± 0.05	**3.90E-04**
**VSD**			
Annulus	0.21 ± 0.01	0.20 ± 0.01	**3.99E-05**
Supero-temporal	0.22 ± 0.00	0.21 ± 0.00	**3.10E-04**
Supero-nasal	0.22 ± 0.01	0.22 ± 0.01	0.45
Infero-temporal	0.22 ± 0.01	0.21 ± 0.01	0.01
Infero-nasal	0.22 ± 0.01	0.22 ± 0.01	0.49
**VCI**			
Annulus	9751.0 ± 437.34	9201.66 ± 551.87	**5.80E-04**
Supero-temporal	2342.77 ± 113.22	2282.22 ± 161.53	0.20
Supero-nasal	2382.01 ± 144.87	2381.79 ± 222.50	0.49
Infero-temporal	2362.61 ± 164.20	2284.16 ± 131.89	0.08
Infero-nasal	2476.66 ± 148.83	2351.54 ± 141.39	0.03
**VPI**			
Annulus	0.46 ± 0.01	0.43 ± 0.02	**1.15E-07**
Supero-temporal	0.45 ± 0.01	0.43 ± 0.01	**2.27E-05**
Supero-nasal	0.45 ± 0.01	0.44 ± 0.01	0.36
Infero-temporal	0.45 ± 0.01	0.42 ± 0.01	**1.41E-05**
Infero-nasal	0.46 ± 0.01	0.45 ± 0.01	0.2
**VDI**			
Annulus	17.23 ± 0.40	16.76 ± 0.41	**1.20E-04**
Supero-temporal	18.24 ± 0.55	17.92 ± 0.43	0.14
Supero-nasal	18.45 ± 0.76	17.87 ± 0.56	0.03
Infero-temporal	18.12 ± 0.67	17.88 ± 0.54	0.23
Infero-nasal	18.07 ± 0.66	17.88 ± 0.44	0.3

Bold values denote statistical significance at the *p* < 0.05 level.

To facilitate data visualization, we generated a VAD heatmap, which is superimposed on top of the threshold vessel image, to help visualize regions of microvascular dropout. In both patients 1 and 2, the VAD heat map (Figures 3D, G) nicely corresponded well with visual field loss (Figure 3A), RNFL thinning (Figure 3E) and GCC thinning (Figure 3H).

### Hierarchical clustering analysis of disk and macular OCT and OCTA data showed 2 distinct groups

We performed quadrant/sectoral analysis of OCT and OCTA parameters in order to ask whether there is preferential regional impact of NAION in optic nerve and macula in NAION. Around the optic nerve, we analyzed six sectors corresponding to the Garway-Heath map (26) (ST: supero-temporal, SN: supero-nasal, IT: infero-temporal, IN: infero-nasal, N: nasal, T: temporal) and four peripapillary quadrants corresponding to the four quadrants on OCT RNFL (S: superior, I: inferior, N: nasal, T: temporal) (Figure 2). For example, the peripapillary VAD in the superior quadrant is called “d.VAD.S” and the disk VSD in the superior quadrant is called “d.VSD.S,” and they correspond with inferior visual field on static perimetry. At the macula, we analyzed four cross quadrants that correspond with four visual field quadrants and six macular sectors corresponding to the macular GCC analysis (ST: supero-temporal, SN: supero-nasal, IT: infero-temporal, IN: infero-nasal, N: nasal, T: temporal). For example, the VAD in the supero-nasal macula cross quadrant is called “m.VAD.SN” and this correlates with inferior GCC and inferior temporal visual field.

**FIGURE 4:**
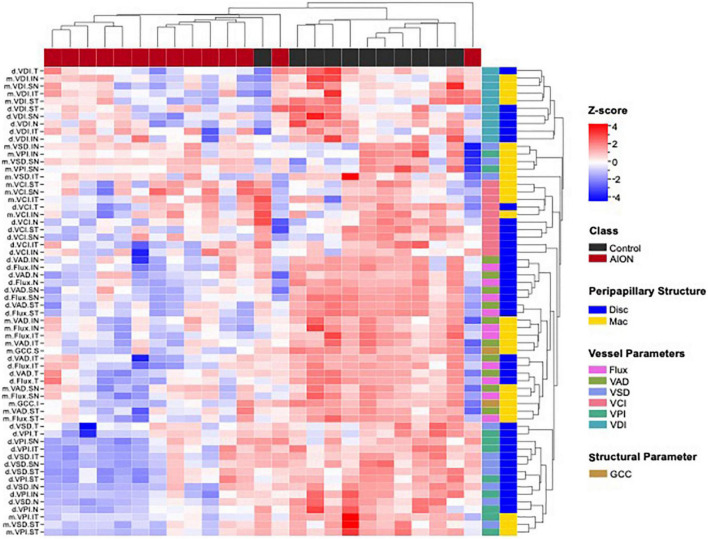
Hierarchical clustering of optic disk and macular optical coherence tomography (OCT) and optical coherence tomography angiography (OCTA) measurement (vertical dendrogram) for all eyes (horizontal dendrogram) showed that data clustered into two distinct groups. Cluster map shows that patients generally showed lower values for each measurement as compared to controls, which corresponds to the deterioration of retinal structure. In the annotation to the right of the heatmap, the colors are defined by the legends on the far right. Disk, optic disk; GCC, ganglion cell complex; Mac, macular; VAD, vessel area density; VCI, vessel complexity index; VD, vessel diameter; VPI, vessel perimeter index; VSD, vessel skeleton density.

We performed hierarchical clustering of all regional parameters for peripapillary and macular OCT and OCTA measurements (vertical dendrogram) for all eyes (horizontal dendrogram). There were two clear clusters: chronic NAION and control eyes. Overall, the NAION eyes have lower OCT and OCTA measurements than controls (Figure 4). Within the OCT and OCTA measurements in the vertical dendrogram, VDI and VCI are clustered together on the top, the VAD and flux are clustered together in the middle, and the VSD and VPI are clustered on the bottom. The top section with VCI and VDI show a greater variability in the data among both chronic NAION and control clusters than the remaining middle and bottom clusters. There are different patterns of OCT and OCTA changes within the NAION eyes. For example, some NAION eyes have relatively low disk and mac measurements, while some NAION eyes have low disk measurements but relatively preserved mac measurements, and some have relatively preserved disk measurements but relatively low macular measurements.

### Chronic NAION eyes with visual field loss show strong optic disk correlation and moderate macular correlation with OCTA measurements

**FIGURE 5:**
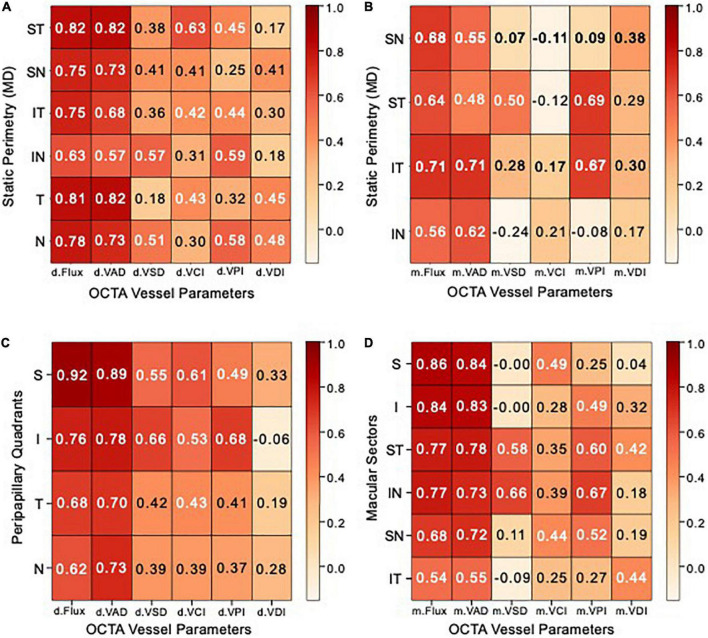
Peripapillary and macular heatmaps showing Spearman correlation matrices. **(A,B)** Heatmap showing Spearman correlation coefficients between optical coherence tomography angiography (OCTA) vessel parameter measurements (X–AXIS) and static perimetry mean deviation (Y–AXIS) of **(A)** peripapillary Garway-Heath sectors, **(B)** macular cross-quadrants. **(C,D)** Heatmap showing Spearman correlation coefficients between OCTA vessel parameters (X–AXIS) and optical coherence tomography (OCT) measurements (Y–AXIS) of **(C)** peripapillary RNFL quadrants, **(D)** macular ganglion cell complex (GCC) sectors. Correlation coefficients between 0.7–1, 0.3–0.7, and 0–0.3 are rated as high, moderate, and low, respectively.

We performed Spearman correlation tests in order to determine which of the six peripapillary and macular OCTA parameters best correspond with visual field MD in each of the peripapillary sectors and macular quadrants (Figure 5). We defined r > 0.7 as high correlation, r between 0.3 and 0.7 as moderate correlation and r < 0.3 as low correlation. Of the 12 OCTA measurements (VAD, VSD, VCI, VPI, VDI, and flux), peripapillary and macular flux and VAD were most highly correlated with MD (r = 0.57–0.82) and the peripapillary measurements were more correlated with MD than macular measurements.

In the peripapillary region, VAD and flux are most highly correlated in the supero-temporal and temporal region with MD (r = 0.81–0.82). The vast majority of peripapillary VSD, VCI, and VPI are moderately correlated with MD (r = 0.3–0.65), while VDI has moderate to low correlation with MD (r = 0.21–0.46) (Figure 5A). In the macular region, VAD and flux are highly correlated in the infero-temporal region (r = 0.71) and moderately correlated in the remaining cross quadrants (r = 0.48–0.68). Macular supero-temporal and infero-temporal VPI were moderately correlated with MD (r = 0.67–0.69) while all other measurements and regions were of relatively low correlation with MD (Figure 5B).

### Chronic NAION eyes show high structural correlation of optic disk of superior quadrant

We performed Spearman correlation tests in order to determine which of the six peripapillary and macular OCTA parameters best correspond with OCT thickness measurements in each of the peripapillary quadrants and macular sectors (Figures 5C, D). Of the 12 OCTA measurements (VAD, VSD, VCI, VPI, VDI, and flux), peripapillary and macular flux and VAD were most highly correlated with OCT thickness (r = 0.7–0.92) and similar to visual field MD, the peripapillary measurements were again more correlated with OCT thickness than macular measurements.

In the peripapillary region, VAD and flux are the most correlated in the superior quadrant to RNFL OCT thickness (r = 0.92). Peripapillary VSD, VCI, and VPI are moderately correlated with OCT (r = 0.37–0.68), while VDI has moderate to low correlation with OCT (r = –0.06 to 0.33) (Figure 5A). In the macular region, VAD and flux were most correlated in the superior sectors than remaining sectors, consistent with peripapillary OCTA measurements. Macular supero-temporal and infero-nasal VPI were moderately correlated with OCT (r = 0.6–0.67) while all other measurements and regions were of relatively low correlation with MD (Figure 5B).

### ANOVA results report greater peripapillary disparity than macular measurements

We compared peripapillary and macular OCT and OCTA measurements using our ANOVA results and graphed the mean whole annulus OCTA values for the two prominent OCTA (VAD and flux) parameters, as well as the OCT measurements (RNFL and GCC) in point plots, for chronic NAION and control eyes. The slope of this point plot illustrates the difference between the optic disk and macula parameters, where the steeper the slope the greater the difference and a more perfused optic disk region. We then reconstructed a line plot with the same slope as the control by aligning the mean macular value with that of the NAION group (labeled “projected NAION”). This line highlights the difference in the ratio of disk to macula values to compare OCTA parameters (VAD and flux) around the optic disk and the macula of chronic NAION patients versus controls; as a control with the same macula value is projected to have a higher disk value than what is graphed for the NAION group. The same was done for OCT measurements for comparing the RNFL and GCC of chronic NAION eyes and controls.

**FIGURE 6:**
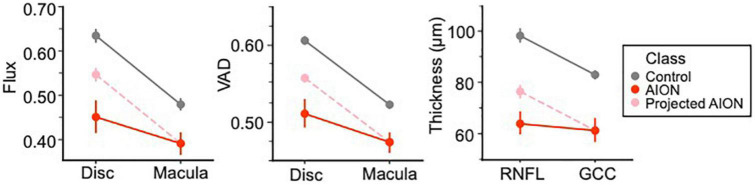
ANOVA results differentiating control and chronic non-arteritic anterior ischemic optic neuropathy (NAION) patients. The results showed that chronic NAION patients’ peripapillary measurements [i.e., retinal nerve fiber layer (RNFL) thickness, disk vessel area density (VAD) and flux measurements] were lower than what we expect based on controls with the same macula values [ganglion cell complex (GCC) thickness, VAD and flux measurements for annulus masks]. We predict this is evidence for the peripapillary structure being “hit twice” in chronic NAION.

Using a two-way factorial mixed ANOVA, we compared the annulus flux measurements of disk and mac structures between the patient and control groups. Assumption of sphericity was not violated as within-subjects variable (structure) had two levels (disk and macula). Levene’s test indicated that group variances were not heterogeneous (disk: *p* = 0.335, mac: *p* = 0.386). There was a significant main effect of group, where the OCTA measurements of controls (gray line on point plot) were greater than that of NAION patients [F(1,45) = 133.74, *p* < 0.001, partial η^2^ = 0.748]. Similarly, results revealed a significant main effect of structure on flux measurements [F(1,45) = 262.99, *p* < 0.001, partial η^2^ = 0.854], where the disk flux measurements were higher than that of the macula. Furthermore, the ANOVA revealed a significant interaction between class and structure [F(1,45) = 49.28, *p* < 0.001, partial η^2^ = 0.523]. This is driven by a greater difference between the VAD measurements of disk and mac in the control group as compared to the NAION group (Figure 6).

We also ran a two-way factorial mixed ANOVA comparing the mean annulus VAD measurements of disk and mac structures for NAION patients and control subjects. There was a significant main effect of structure [F(1,46) = 285.32, *p* < 0.001, partial η^2^ = 0.861], where disk VAD measurements were higher than that of macula. As Levene’s test revealed that assumptions of homoscedasticity were violated (disk: *p* = 0.001, mac: *p* = 0.002), we analyzed the main effect of class using the Welch’s adjusted F ratio. Both disk [Welch’s F(1, 24.34) = 148.54, *p* < 0.001] and mac [Welch’s F(1, 21.99) = 50.32, *p* < 0.001] were significantly different in VAD measurements between NAION and controls. There is also a significant interaction between class and structure [F(1,46) = 42.99, *p* < 0.001, partial η^2^ = 0.483]. Like flux, this is due to a greater difference between disk and mac VAD measurements in the control group than the NAION group.

We ran a third two-way factorial mixed ANOVA comparing the average RNFL and GCC thickness from OCT between patient groups. There was a significant main effect of structure [F(1,42) = 53.85, *p* < 0.001, partial η^2^ = 0.562] and class [F(1,42) = 185.31, *p* < 0.001, partial η^2^ = 0.815]. Similar to OCTA results, there was a significant interaction between structure and class [F(1,42) = 21.10, *p* < 0.001, partial η^2^ = 0.334], where RNFL and GCC thickness in NAION patients were consistently thinner than controls, but this disparity is greater in RNFL thickness as compared to GCC.

### Logistic regression with LASSO regularization identifies OCTA parameters to predict NAION

We performed separate logistic regression models with LASSO regularization and identified that whole annulus VAD, VCI, and VDI are the best peripapillary OCTA parameters and VAD and flux are the best macular parameters that statistically best predict disease status (Table 4). Topographical analysis using the logistic regression model reveals following optic disk parameters according to the modified Garway Heath map sections as the best parameters for predicting NAION: Flux supero-temporal (Flux.ST), VCI temporal (VCI.T), VPI inferior (VPI.I), and VPI infero-nasal (VPI.IN). The best macular cross-quadrant OCTA parameters identified by the same model reveals VAD infero-temporal (VAD.IT), Flux infero-temporal (Flux.IT), VPI supero-temporal (VPI.ST), and VPI infero-temporal (VCI.IT) as the best macular parameters that statistically best predict disease status (Table 4).

**TABLE 4:** Logistic regression results with the most predictive annulus and regional optical coherence tomography angiography (OCTA) parameters of peripapillary and macular regions of non-arteritic anterior ischemic optic neuropathy (NAION) disease status.

Annulus		Regional	
Optic disk	Macula	Optic disk (Garway Heath Sectors)	Macula (Cross-quadrants)
VAD	VAD	Flux_ST	Flux_IT
VCI	Flux	VCI_T	VAD_IT
VDI	–	VPI_I	VPI_IT
–	–	VPI_IN	VPI_ST

## Discussion

Optical coherence tomography and OCTA are invaluable in the clinical setting for assessing NAION patients because they produce vital structural and vascular measurements of the optic nerve and the macula to serve as biomarkers for predicting visual outcome. OCTA is a novel, non-invasive technology that is increasingly utilized in assessing patients with optic neuropathies like glaucoma and in neuro-ophthalmic causes of optic neuropathies (32–36). OCTA imaging reflects blood flow changes that may reflect functional impairment prior to irreversible structural thinning. We performed a detailed analysis on OCTA parameters with corresponding OCT and visual field MD measurements per modified Garway-Heath map. To our knowledge, we have analyzed more whole eye and regional OCT and OCTA parameters than prior studies, totaling 154 in number, to allow for the most comprehensive analysis of both peripapillary and macular regions of chronic NAION patients versus controls. While most studies report whole eye correlation between OCTA and supplementary OCT or static perimetry measurements, our study also reports a comprehensive regional correlation of parameters for the optic disk and the macula.

### VAD most correlative to static perimetry and OCT measurements

We analyzed six OCTA parameters and found that VAD and flux were the most correlative parameters to visual field MD and OCT thickness in the peripapillary region. Our custom algorithm provides the advantage of removing large vessels that can false-negatively affect superficial vessel density and quantifies only the capillaries that reflect optic nerve perfusion. While VAD, the most quantified OCTA parameter, has previously been shown to be significantly decreased in the peripapillary region in NAION (16, 19, 37–46), our study shows for the first time that VAD and flux are most correlated among the other vascular parameters. We also show that flux closely resembles VAD in correlating with RNFL thickness and Garway-Heath visual field MD measurements. VAD is also predictive of patient status in NAION and can be the single best microvascular parameter. The VAD heatmap generated by our algorithm, as shown in Figure 3D. Is a superior way to visualize the two-dimensional data that can aid in the clinical assessment of NAION patients. In addition to VAD, peripapillary VCI and VDI are important biomarkers to predict NAION, which has not been revealed in previous studies.

While OCTA studies typically quantify vascular parameters surrounding the optic disk alone, our findings not only reveal that macular VAD and flux are the most decreased vascular parameters in chronic NAION patients, but also that VAD and flux were most correlated with visual field and OCT measurements in the macular region, perhaps making them the most important macular measures of neurodegeneration. Our study is the first to report that flux is closely correlated with VAD in the macular region among other OCTA parameters and that macular SCP vascular changes closely resemble the peripapillary microvasculature pattern. This macular correlation with OCT and visual field MD measurements has not previously been described in OCTA studies. Very few OCTA studies have analyzed microvascular density in the macular region in NAION patients, but the results showed different patterns of changes in microvasculature (43, 47–51). Some OCTA studies without large vessel removal report no significant changes in the macular microvasculature in a small subset of NAION patients (52). Our macular analysis reports that macular VAD and flux are valuable measurements in clinical assessment of NAION, which was reported in a small number of studies without large vessel removal that report a decrease in macular vessel density alone (47, 52, 53). Our study suggests that flux is not only a complementary parameter to the conventional VAD, but may also be an equally sensitive measure of changes in capillary perfusion as VAD. Fard et al. (54) compared whole image macular microvasculature density in NAION and primary open angle glaucoma (POAG) patients and reported that NAION patients had lower macular vessel density than controls but not lower than of POAG patients. Our study demonstrates that one can measure mean macular VAD and flux to help in the clinical assessment of chronic NAION patients and to predict visual outcome.

### Regional changes in superficial microvasculature

In addition to our whole annulus data, our study provides the most detailed regional analysis of peripapillary and macular regions in NAION eyes compared to controls. Our data showed that the VAD and flux, the most important vascular parameters, was more affected in the superior and temporal peripapillary regions than the inferior regions, with corresponding loss of the inferior >superior visual field. This is in support of the common characteristics of NAION, which manifests as superior OCT thinning and predominantly inferior and nasal visual field defects (55–58). Our study reports that statistically, OCTA parameters such as VAD, flux, and VCI best predict disease status.

When comparing temporal and nasal peripapillary regions based on the modified Garway-Heath map, our data reveals that the temporal region is more greatly affected and better correlated with visual field MD and RNFL OCT thickness. This is in support of OCTA studies without large vessel removal which reported that temporal peripapillary vessel density were more affected and significantly correlated with logMAR VA (37, 58), while others report a reduction in SCP vessel density in all peripapillary regions except the nasal quadrant (37, 59, 60). Our regional analysis around the optic disk show that the superior and temporal peripapillary region were most important and correlated with OCT and visual field measurements, making them potentially the most predictive measurements in early NAION for prognosticating visual field loss.

In macular analysis, our study reveals that OCTA VAD and flux in the temporal macula were most correlated with visual field MD measurements. Our correlation matrices show that OCTA VAD and flux highly correlate in the superior sectors with the GCC sectoral OCT thickness. Of the limited OCTA studies that have analyzed the macular region, there is a lack of agreement in the characteristic pattern of regional changes in the microvasculature (43, 48–51). For example, Liu et al. (43) showed a lower SCP vessel density in the superior perifoveal region in NAION patients and Moon et al. report that the SCP of nasal macular region is more affected and correlate significantly with a poor visual outcome (50). To our knowledge, we are the first to report macular parameters in addition to VAD and flux such as VPI in the infero-temporal and supero-temporal quadrants as potentially important regional macular parameters. Overall, our macular analysis is in close agreement with the regional peripapillary changes in microvasculature but in a lesser degree of severity, which has not been reported in the literature and potentially making them the most predictive regional measurements for vision loss in NAION.

Our logistic regression results inherently encompass the key OCTA findings of our study by confirming the importance of VAD in the prediction of NAION in both the peripapillary and macular region and the importance of other vascular biomarkers such as VCI, VDI, and VPI. In addition, flux is also, perhaps equal in potential as VAD, is an important predictive macular biomarker of NAION. The logistic regression also supports the regional effects on capillary perfusion in NAION by emphasizing the superior and/or temporal regions as well as the same key regional OCTA parameters mentioned in addition to VAD and flux.

Among all the parameters we examined, OCTA VAD, flux, and OCT thickness were consistently more affected in the peripapillary region than in the macular region, potentially because the peripapillary region is primarily affected from the acute stages of NAION. In the acute stage of NAION, vision loss and RNFL thinning on the visual field and OCT, respectively, is thought to arise from the primary ischemic insult due a decreased perfusion to the optic nerve. Over time, chronic NAION leads to secondary loss of SCP density in the peripapillary and true secondary loss in the parafoveal region as a result of retrograde degeneration of intraretinal axons and ganglion cell bodies, leading to further thinning of the OCT measurements (2, 61–63). This is demonstrated in many parts of our analysis. Our two-way factorial mixed ANOVA of VAD and flux for the optic disk and the macula reveal a greater disparity of OCTA measurements on the optic disk than the macula between NAION patients and controls. This disparity in OCTA findings between optic disk and macular are supplemented by our two-way factorial ANOVA for OCT measurements (RNFL and GCC) of both regions by reporting a greater difference in RNFL thickness between controls and NAION patients than that of GCC thickness. The agreement in our ANOVA results collectively tie in with our Spearman correlation analysis which reveal higher correlation coefficients overall for OCTA parameters in the peripapillary region than the macular region to both visual field MD and OCT thickness measurements. The primary ischemic insult to the optic nerve results in RNFL thinning that is exacerbated during retrograde degeneration of the intraretinal axons in the parafoveal region resulting in secondary loss of GCC and RNFL. While the macula is less severely affected, its affected pattern resembles and closely correlated with the optic nerve from the retrograde degeneration. Our findings clearly support the use of peripapillary and parafoveal OCTA analysis in support of OCT and visual field measurements to reflect primary and secondary ischemic insults in chronic NAION.

There are several limitations in this study. This is a small, retrospective study without longitudinal data points, which is critical for understanding the evolution of acute to chronic stage of NAION. This should be addressed in further large-sample, prospective studies. Our OCTA findings only address the changes in the superficial microvasculature and not the deep plexus, which may also be affected by chronic NAION. Another limitation to the study is the difference in the dimensions of the region of interest used to calculate peripapillary OCT RNFL and OCTA. While the macular GCC sectors vertical and horizontal diameters of the ellipsoid scan in OCT match the OCTA ellipsoid sector scan, there are differences in the peripapillary OCT RNFL and OCTA dimensions. The sd-OCT RNFL was segmented on a circular B-scan with 3.46 mm diameter centered on the optic disk. The OCTA of the SCP microvasculature was measured using en face 6 × 6 images of an annulus with an outer radius of 2.4 mm (outer diameter of 4.8 mm) and inner radius of 0.5 mm (inner diameter of 1.0 mm) centered around the optic nerve. This may result in small differences in the quadrant analysis of the peripapillary region between OCT and OCTA.

## Conclusion

In conclusion, our study demonstrates that both OCT and OCTA measurements are important in the assessment of injury in NAION. Peripapillary and macular VAD and flux showed the strongest correlation with static perimetry mean deviation and OCT RNFL and GCC thickness. Multiple OCTA parameters in addition to VAD and flux, particularly in the superior and inferior temporal region, are the most predictive of having NAION. OCTA and complementary OCT measurements were more reduced in the peripapillary region than that of the macular region in chronic NAION, consistent with the peripapillary area as the primary site of injury and retrograde macular degeneration.

## Data availability statement

The raw data supporting the conclusions of this article will be made available by the authors, without undue reservation.

## Ethics statement

The studies involving human participants were reviewed and approved by Institutional Review Board by Stanford University. The patients/participants provided their written informed consent to participate in this study.

## Author contributions

SP, MY, and YL contributed to the conception or design of the work and made acquisition, analysis, and interpretation of data for the work. GZ, YC, and RW provided algorithm that was used for the analysis of OCTA data. SP, MY, GZ, YC, RW, and YL drafted the manuscript and critically reviewed the manuscript for important intellectual content. All authors agreed to be accountable to all aspects of this work ensuring that questions related to the accuracy or integrity of any part of the work are appropriately investigated and resolved and contributed to the article and approved the submitted version.

## Footnotes

1
http://www.python.org
